# SHEPHARD: a modular and extensible software architecture for analyzing and annotating large protein datasets

**DOI:** 10.1093/bioinformatics/btad488

**Published:** 2023-08-04

**Authors:** Garrett M Ginell, Aidan J Flynn, Alex S Holehouse

**Affiliations:** Department of Biochemistry and Molecular Biophysics, Washington University School of Medicine, 660 South Euclid Avenue, Saint Louis, MO 63110, United States; Center for Biomolecular Condensates, Washington University in St. Louis, 1 Brookings Drive, Saint Louis, MO 63130, United States; Department of Biochemistry and Molecular Biophysics, Washington University School of Medicine, 660 South Euclid Avenue, Saint Louis, MO 63110, United States; Center for Biomolecular Condensates, Washington University in St. Louis, 1 Brookings Drive, Saint Louis, MO 63130, United States; Department of Biochemistry and Molecular Biophysics, Washington University School of Medicine, 660 South Euclid Avenue, Saint Louis, MO 63110, United States; Center for Biomolecular Condensates, Washington University in St. Louis, 1 Brookings Drive, Saint Louis, MO 63130, United States

## Abstract

**Motivation:**

The emergence of high-throughput experiments and high-resolution computational predictions has led to an explosion in the quality and volume of protein sequence annotations at proteomic scales. Unfortunately, sanity checking, integrating, and analyzing complex sequence annotations remains logistically challenging and introduces a major barrier to entry for even superficial integrative bioinformatics.

**Results:**

To address this technical burden, we have developed SHEPHARD, a Python framework that trivializes large-scale integrative protein bioinformatics. SHEPHARD combines an object-oriented hierarchical data structure with database-like features, enabling programmatic annotation, integration, and analysis of complex datatypes. Importantly SHEPHARD is easy to use and enables a Pythonic interrogation of largescale protein datasets with millions of unique annotations. We use SHEPHARD to examine three orthogonal proteome-wide questions relating protein sequence to molecular function, illustrating its ability to uncover novel biology.

**Availability and implementation:**

We provided SHEPHARD as both a stand-alone software package (https://github.com/holehouse-lab/shephard), and as a Google Colab notebook with a collection of precomputed proteome-wide annotations (https://github.com/holehouse-lab/shephard-colab).

## 1 Introduction

**Figure 1.**
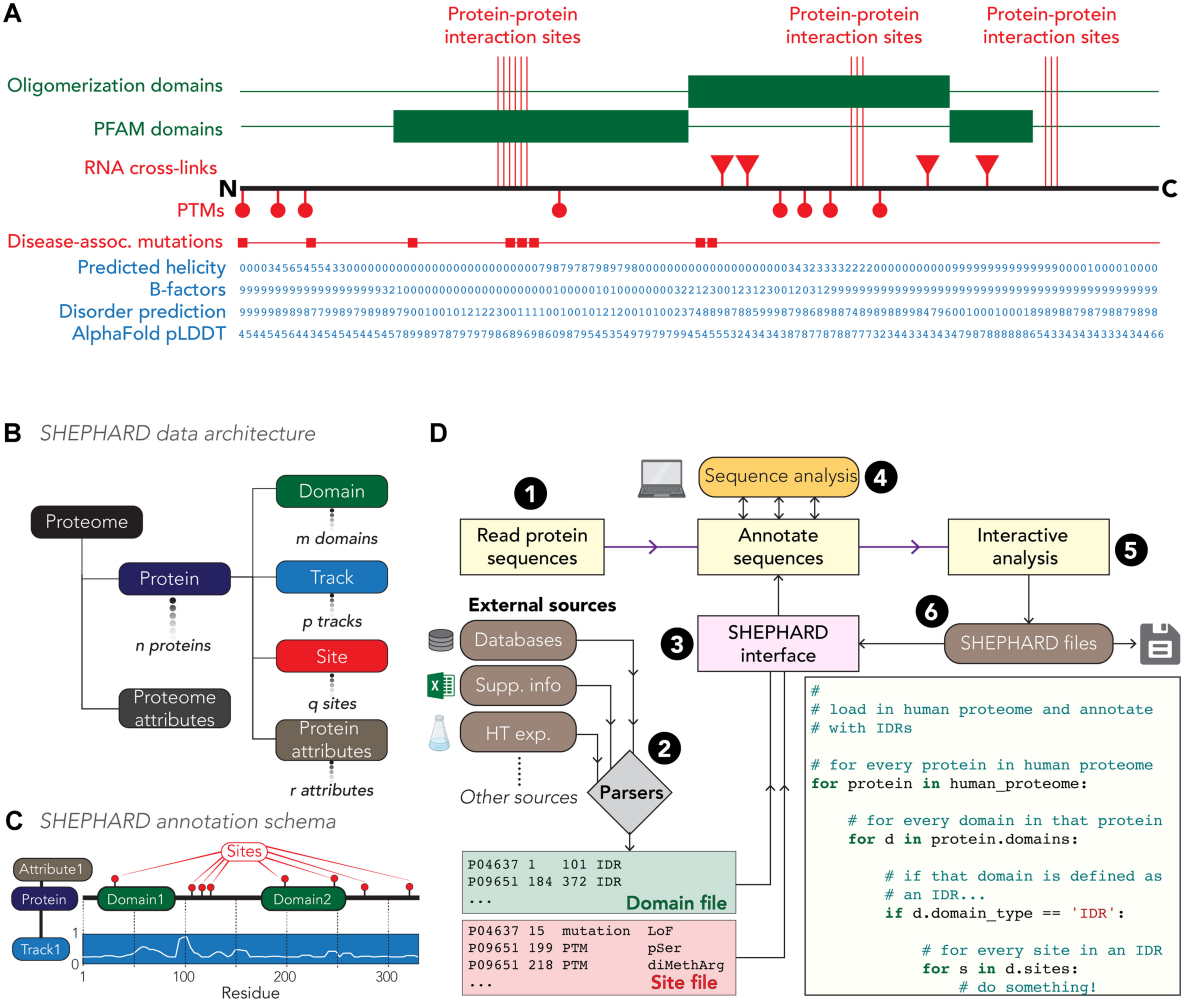
(A) Protein annotations span a range of flavors and types. (B, C) SHEPHARD uses four protein-level annotations, and multiple Proteins are organized in Proteomes. (D) SHEPHARD provides an ecosystem of tools for reading, analyzing, and writing annotations to facilitate syntactically simple analysis of large protein datasets.

Over the last two decades, high-throughput experiments have enabled the acquisition of large datasets that offer insight into biologically important features for thousands of proteins simultaneously (Zhang *et al.* 2014, Huang *et al.* 2015, Riley and Coon 2016, Kinney and McCandlish 2019). When combined with traditional and deep-learning-based computational approaches, proteome-wide annotation enables the generation of high-dimensional data ripe for further analysis (UniProt Consortium 2015, Lindorff-Larsen and Kragelund 2021, Necci *et al.* 2021, Tunyasuvunakool *et al.* 2021, Sapoval *et al.* 2022) (Fig. 1A). If integrated, these datasets can be used to ask large-scale statistical questions on the relationship(s) between different types of annotations. These analyses can generate and test novel hypotheses, extracting additional value from previously published data in ways the original authors may have never anticipated.

Despite remarkable progress in data generation and acquisition, downstream processing and integration are often treated as an afterthought. Proteome-wide datasets that require incredible resources and effort to generate are often deposited in poorly-labeled Excel spreadsheets or hard-to-parse text files. In particular, the ability to cross-reference across many different types of annotations raises several practical challenges, including data cleaning, developing and applying appropriate data structures, and the portability and reliability of analysis code. These issues contribute to a scenario where large-scale analyses are often performed in a relatively ad hoc way. Researchers often invest substantial resources in building robust frameworks. Alternatively, they hack together bespoke, one-off pipelines, which may suffer from integrity, completeness, or reproducibility issues.

We have addressed this challenge by developing a modular and extensible software architecture for large-scale and high-throughput protein sequence analysis. SHEPHARD is a Python-based general-purpose hierarchical framework that facilitates reproducible, reliable, and high-throughput analysis of complex numerical and symbolic protein annotations at proteome-wide scales.

## 2 Materials and methods

SHEPHARD addresses three main challenges in the context of data integration and analysis. First, SHEPHARD enables a clear and syntactically simple programming interface for asking broad, integrative statistical questions across large datasets. Second, SHEPHARD makes it easy to read annotations from external sources and write annotations to files, making it easy to integrate into existing workflows. Third, we provide SHEPHARD as a locally-installable Python package and Google Colab notebooks. These notebooks include various preloaded annotations for the human proteome. Together, SHEPHARD provides features for both seasoned bioinformaticians and novice users.

SHEPHARD stores data in an object-oriented hierarchical format where the base container is a Proteome. Proteomes contain one or more Proteins, and each Protein can be annotated with Domains, Sites, or Tracks (Fig. 1B and C). Proteomes and associated annotations are read in and written out via a set of routines that provides an interface between the outside world and SHEPHARD. To ensure SHEPHARD-associated annotations are easy to generate, read, and interpret, we have introduced a simple tab-separated schema for defining SHEPHARD-associated data. Text-based tab-separated file formats are commonplace in bioinformatics (e.g. BED files), meaning we avoid creating a fundamentally new type of file. Our file implementation stores one annotation per line with specific column definitions, making these files easy to generate using existing bioinformatic scripts or MS Excel, or Google Sheets. Importantly this format can also be opened and easily understood via commonly-used software packages (e.g. MS Excel). In this way, we ensure SHEPHARD-generated data remain accessible even for scientists with a minimal background in computer science. Please see the supporting information for a detailed discussion of the software design principles that underline SHEPHARD.

Having introduced the conceptual and practical features that SHEPHARD addresses, the remainder of this report illustrates the types of integrative questions SHEPHARD makes easy to ask and answer across the human proteome.

## 3 Results

**Figure 2.**
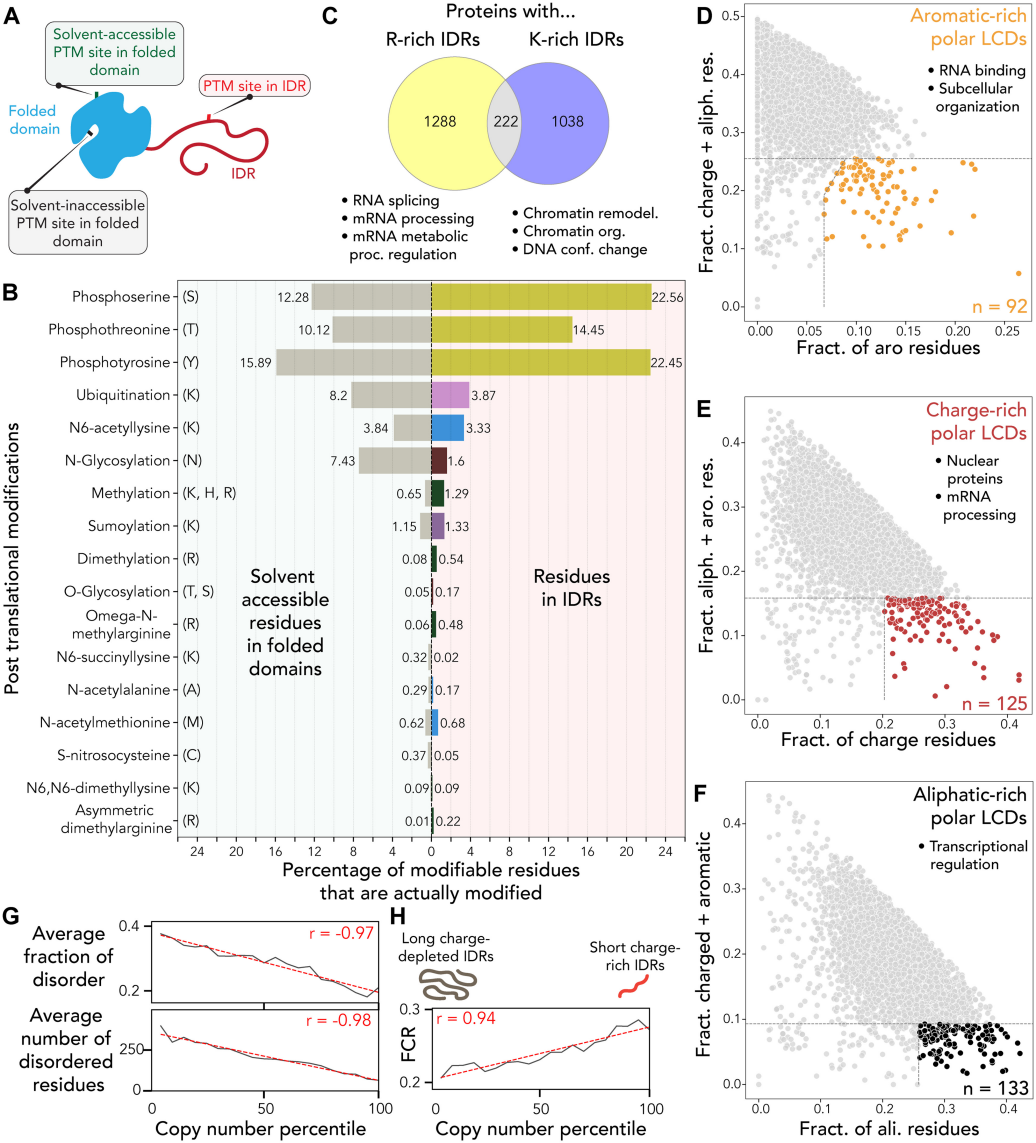
(A) Schematic of different types of PTM site types. (B) After accounting for structural context, residues in IDRs are still preferentially modified by PTMs. (C) Proteins with arginine or lysine-rich IDRs are largely nonoverlapping in function and have distinct nucleic-acid binding preferences. (D, E, F) Proteins with polar-rich low-complexity domains enriched for specific types of chemistry have distinct functional enrichments. Enrichment here reflects being in the top 20% of IDRs by the fraction of the chemistry of interest and also in the bottom 5% of the chemistry being depleted (e.g. in panel D, we identify the proteins in the top 20% of aromatic residues but bottom 5% of charge + aliphatic residues). (G) Highly expressed human proteins tend to be depleted for IDRs (on average) (*P* < 0.0001). (H) IDRs in highly expressed human proteins tend to be more highly charged (*P* < 0.0001).

Intrinsically disordered regions (IDRs) often contain post-translational modification (PTM) sites (Supplementary Tables S1 and S2) (Iakoucheva *et al.* 2004). We wondered if this observation reflects a bona fide preference of modifying enzymes for IDRs. Conversely, this result may be a convolution of sequence composition bias and differences in solvent accessibility for residues in IDRs versus folded domains (Fig. 2A). To answer this, we combined proteome-wide per-residue binding accessibility data based on AlphaFold2 predicted structures with IDR and PTM annotations. These analyses reveal that after correcting for compositional biases and solvent accessibility, many (but not all) types of modifications remained enriched in IDRs, in line with recent work (Fig. 2B) (Bludau *et al.* 2022). For example, around 23% of serine residues in IDRs are phosphorylated compared to just 12% of solvent-accessible serine residues in folded domains. Moreover, most modifications occur in either disordered regions, flexible loops with no defined secondary structure, or alpha helices, suggesting these types of substrates are generally preferred (Supplementary Figs S1 and S2).

While IDRs are often treated as a monolithic class of domains, IDR conformational behavior and function can be influenced or even determined by amino acid sequence composition (Das *et al.* 2015, Zarin *et al.* 2021). As such, we examined functional differences in IDRs enriched for either arginine or lysine, amino acids with different positively charged sidechains. While proteins with arginine-rich IDRs were enriched for RNA binding functions, those with lysine-rich IDRs were enriched for DNA binding (Fig. 2C, Supplementary Tables S3 and S4). This analysis reveals interpretable chemical differences in nucleic-acid interaction preferences, mirroring previous demonstrations of a clear difference in higher-order assemblies driven by arginine or lysine-rich peptides (Boeynaems *et al.* 2019).

Motivated by our results from examining arginine and lysine-rich IDRs, we wondered if distinct flavors of polar-rich low-complexity domains (pLCDs) might be associated with specific functions. pLCDs are regions in IDRs enriched in polar amino acids (glycine, serine, threonine, glutamine, asparagine, and here also proline) (Millard *et al.* 2020, Gutierrez *et al.* 2022). Given the polar nature of polypeptide backbones, pLCDs present a chemically homogenous scaffold that could be punctuated by other types of orthogonal chemistry. The kinds of sequence chemistry encoded by the remaining natural twenty amino acids can be broadly categorized as aliphatic, aromatic, or charged (Martin and Holehouse 2020). We identified pLCDs and filtered for those depleted in two of these three types of chemistry but enriched for the third. This revealed a clear difference in the types of proteins associated with chemically-distinct pLCDs (Fig. 2D–F). Aromatic-rich pLCDs (*n* = 89) are enriched for RNA binding proteins as well as proteins involved in cellular structural components (intermediate filaments, nuclear pore complex, adhesion junction proteins) (Supplementary Tables S5 and S8). Charge-rich pLCDs (*n* = 106) are largely enriched in nuclear proteins across a range of functions (notably RNA splicing and RNA processing) (Supplementary Tables S6 and S9). Finally, aliphatic-rich pLCDs (*n* = 117) are enriched in proteins associated with transcriptional regulation (including transcription factors and chromatin binding proteins) (Supplementary Tables S7 and S10). Our results are consistent with a model whereby IDR chemical context can prime IDRs for specific functions.

Finally, prompted by prior work linking protein disorder to dosage-dependent toxicity, we wondered how the presence of disordered regions might correlate with protein abundance(Bolognesi *et al.* 2016). To our surprise, we observed a strong negative correlation between human protein copy number obtained by quantitative mass spectrometry and disorder (Fig. 2G). Encouragingly, this trend was reproduced across several other organisms with data from independent experiments (Supplementary Figs S3 and S4). Intriguingly, across the human and yeast proteomes, we observed a strong correlation between how charged an IDR is and the copy number (Fig. 2H). IDRs in highly-abundant human proteins tend to be more highly charged. As such, we speculate that solubility—as determined by the fraction of charged residues—may play a role in defining the fitness advantage/defect associated with the presence of a given IDR. Our simple interpretation from these data is that long uncharged IDRs are generally more harmful than short, charged IDRs.

## 4 Discussion

SHEPHARD enables integrative proteome-wide bioinformatic analysis. In the case of expert users, it provides a route for developing complex bioinformatic pipelines that take care of several key steps required for protein-based bioinformatics. In the case of novice users, pregenerated proteome-wide annotations are made accessible via Google Colab notebooks, enabling people to perform integrative bioinformatics in their web browser. To illustrate the types of analyses SHEPHARD enables, we have provided examples where annotations from AlphaFold2, post-translational modification experiments, predicted disorder, sequence chemistry, and protein abundance data are seamlessly integrated. These analyses reveal new insights into the structural context of PTMs, the role of sequence chemistry in the function of disordered regions, and the relationship between disorder and protein abundance. In summary, SHEPHARD offers a convenient resource for those working on understanding protein-function relationships at scale in a distributable, reproducible, and reliable manner.

SHEPHARD offers a standard programmatic interface such that similar or identical analysis code can be used to analyse completely different datasets. It also provides important behind-the-scenes consistency and sanity checking to help catch simple errors, malformatted data, or inconsistent annotations. These features enable researchers to dedicate their time to developing innovative analysis approaches instead of worrying about data parsing and sanity checking. In addition, we have precomputed a large number of annotations for the human proteome and written detailed Google Colab notebooks, such that with a rudimentary understanding of the Python programming languages, scientists can conduct large-scale proteome-wide bioinformatic analysis with relative ease, either online or by downloading our precomputed annotations. To clearly illustrate the value added by SHEPARD, we provide a brief walkthrough comparing the steps needed to complete the analyses enabled by these precomputed annotations in the supporting information.

SHEPHARD is also designed to be helpful for both computational and noncomputational researchers. In particular, we wanted to make it easy for computational scientists to share their data in a way that is easily accessible to scientists with no background in computer science at all. Compiled or highly structured data formats (e.g. GFF, BED, JSON) have many advantages from the perspective of information theory, metadata, or extendability. However, these formats can suffer from the fact that they are often uninterpretable to scientists without the requisite technical expertise. To make this concrete, a goal for SHEPHARD was to ensure the file formats that are read in or written out can be opened by “clicking on an icon” and using software most users already have access to (e.g. Microsoft Excel). With this in mind, SHEPHERD-compliant data files are defined in a simple, consistent, and well-defined tab-separated data format. This means after complex analysis pipelines, input and output data can be shared as supplementary information in a format that almost anyone can open and understand. It also ensures that computational and noncomputational scientists consistently work with the same data. The decision to implement a specific schema for TSV files that hold Tracks, Sites, and Domains (as opposed to using an existing bioinformatics file format) reflects the balance of ensuring SHEPHARD files are easily readable and writable and avoiding overloading an existing file format for data it was not designed to accommodate (e.g. protein information into a BED file).

As a final note, from a reproducibility standpoint, SHEPHARD enables complex bioinformatics pipelines to be written in a few lines of code. The ability to easily re-analyze changing datasets via a consistent software interface will enable work published today to be re-evaluated a decade from now without the need to change the actual pipeline. From a software engineering standpoint, SHEPHARD was designed to possess an internally consistent data architecture combined with loosely-coupled interfaces and APIs. This design strategy means SHEPHARD does not make any assumptions about the outside world, avoiding challenges introduced by changing dependencies or deprecation of external libraries. While not the most glamorous of topics, software longevity is a critical feature in an ever-changing ecosystem. SHEPHARD was deliberately designed to ensure maintainability and extensibility did not come at the expense of tight coupling to other software tools or data formats.

## Supplementary Material

btad488_Supplementary_Data

## Data Availability

All data and code used to perform all analyses in this report can be found at https://github.com/holehouse-lab/supportingdata/tree/master/2022/ginell_2022. SHEPHARD documentation can be found at https://shephard.readthedocs.io/, and Colab notebooks can be found at https://github.com/holehouse-lab/shephard-colab. The SHEPHARD itself can be downloaded and installed from PyPI (https://pypi.org/project/shephard/) or GitHub (https://github.com/holehouse-lab/shephard). Precomputed annotations for many model proteomes, including proteome-wide annotations and 3rd-party studies [e.g. data from reference (Tsuboyama *et al.* 2023)], are available at https://github.com/holehouse-lab/shephard-data.
