# Supplementary material for: SHEPHARD: a modular and extensible software architecture for analyzing and annotating large protein datasets
Source: Bioinformatics. 2023 Aug 4;39(8):btad488. doi: 10.1093/bioinformatics/btad488 (PMC10423030; doi:10.1093/bioinformatics/btad488)
Supplement: btad488_Supplementary_Data [file btad488_supplementary_data.docx]

**SUPPLEMENTARY INFORMATION:**

**SHEPHARD: a modular and extensible software architecture for analyzing and annotating large protein datasets**

Garrett M. Ginell, Aidan J. Flynn, Alex S. Holehouse

Department of Biochemistry and Molecular Biophysics, Washington University School of Medicine, St. Louis, MO, USA

**Correspondence**: alex.holehouse@wustl.edu

1. SHEPHARD IMPLEMENTATION AND ARCHITECTURE

***SHEPHARD DESIGN DECISIONS***

SHEPHARD is written in the Python programming language (<https://www.python.org/>) with limited core dependencies. Specifically, SHEPHARD requires Numpy^1,2^, a well-established library for numerical computing in Python, and protfasta, a high-performance FASTA parser developed explicitly for large protein datasets^3^. Beyond these two external packages, our goal is to limit hard dependencies to core components of the Python scientific computing stack, keeping installation easy and lightweight.

SHEPHARD is built as a set of loosely coupled components. At its core, SHEPHARD contains a set of hierarchically-linked and self-referential data storage objects. These objects are **Proteomes**, **Proteins**, **Tracks**, **Domains**, and **Sites**. Each Proteome object stores zero or more Protein objects. Each Protein object stores zero or more Track objects, Domain objects, or Site objects. Tracks, Domains, and Sites are all aware of the Protein they originate from, such that analysis across all Sites, Tracks, or Domains can be performed as easily as it can be done over every Protein. In addition, Proteomes, Proteins, Sites, and Domains can have attributes associated with them. These attributes are key:value pairs where the key defines an attribute name, and the value can be almost anything. Because arbitrary attributes can be annotated for all object types except Tracks, it is possible to extend annotations of a protein to whatever specific type of analysis is of interest. For example, SHEPHARD can be used to work with protein simulation data, where Protein attributes map to trajectories, or to protein cross-linking datasets, where site attributes include lists of cross-linked Sites (residues) from other proteins to construct interactome networks.

SHEPHARD data objects can be added and removed as analysis is performed. Tracks, Sites, and Domains can be added and removed from Proteins, and Proteins can be added or removed from Proteomes. The dynamism enables changes to data structures during analysis, facilitating complex unidirectional pipelines that generate *de novo* annotations, prune datasets, or augment existing annotations.

A major advantage of casting protein annotations into four distinct types (Domains, Sites, Tracks, or Attributes) is that essentially identical code can often be re-used to analyze very different questions. By presenting a common style of interface for each of these objects, analogous design patterns can be learned for analyzing Sites and then applied to Domains (for example).

***Sequence indexing***

Python indexes from 0 and is exclusive (i.e., in so-called “slice syntax,” the string “**abcde**” sliced as **abcde[1:2]** would return “**b**”). However, in biology (and in natural counting), we typically index from 1 and use an inclusive convention; that is, a domain that straddles residues 1-20 starts at the first residue (residue 1) and includes the 20^th^ (residue 20). SHEPHARD assumes this second indexing convention - that is, the first residue in a sequence is 1, and requesting subregions of sequences will honor this and include the residue associated with the end position. This decision avoids users needing to perform offset corrections and makes it much easier to relate position in SHEPHARD to real-world residue numbering in sequence annotations. It also means if sequence subregions are to be excised, we provide a **get_sequence_region(start, end)** function in the Protein class.

***Interfaces***

In addition to the core data objects, SHEPHARD uses an **interfaces** module to read and write data to and from core data objects. Interfaces are a general design pattern from software engineering in which a common input format or structure is guaranteed to be readable by the associated software. In effect, an Interface provides a contract between the inner code of SHEPHARD and the outside world. The use of an interface module means that to load *any* type of data into SHEPHARD, the user need only transform their data into a datatype that is compliant with the interface. The alternative approach would be to build custom parsers for each data type, which places the burden of file parsing in the hands of the software developer and away from the expertise associated with the data. This is neither sustainable nor practical.

***Application programming interfaces (APIs)***

In addition to the ability to read/write SHEPHARD-compliant annotations, SHEPHARD also provides an application programming interface (API) for interacting with specific external packages or data types. Including an API module allows us to extend SHEPHARD to provide dedicated functionality for specific types of input, output, or analyses that go beyond the core data structures and interfaces. At the time of writing, the APIs include those for working with generic FASTA files and those for working with FASTA files obtained from UniProt. We anticipate adding additional APIs as the need arises.

***Sanity checking***

As a final note, SHEPHARD employs a ‘safety first’ approach to data parsing. That is, by default, operations that would overwrite data or lead to inconsistent behavior raise exceptions (errors). This behavior can be turned off on a case-by-case basis using the **safe** keyword. This design decision helps catch unexpected errors, conceptually flawed data types, or other problematic issues that may otherwise be silently missed.

***UNDERLYING SOFTWARE ARCHITECTURE***

***Data types and data structure***

SHEPHARD implements a hierarchical data structure with datatypes that map to easily-interpretable protein features.

The base object in SHEPHARD is a ***Proteome object***. A Proteome is a collection of zero or more proteins. As well as providing a general organizing collection for Protein objects, Proteins enable easy access to a range of functionality. This includes retrieving all Domains or Sites of a certain type across all proteins and retrieving the list of unique Domain or Site types found in the proteome. Proteome objects are also generators for proteins, meaning in Python syntax, one can iterate across the proteins in a Proteome simply by iterating over a for-loop.

***Protein objects*** possess information on an individual protein. This includes the underlying amino acid sequence, a unique identifier, a protein name, and zero or more annotations with Tracks, Domains, Sites, and Attributes. In addition, proteins contain a set of functions for excise sequence regions and contain the ability to add/remove Tracks, Domains, Sites, or Attributes.

***Track objects*** contain vectorial information that maps to each residue along a protein sequence and can encode either numerical (a values track) or symbolic information (a symbols track). A protein can have multiple tracks, but all tracks must match the sequence length. Tracks could be used to encode per-residue predictions, perform sequence coarse-graining, or encode experimental information in which a single value per residue is generated (e.g., NMR chemical shifts, crystallographic B factors, CryoEM resolution, *etc.*)

***Domain objects*** describe discrete sub-regions within a protein and are defined by a start and end location, as well as a domain type. They are automatically named using a convention of **${domain_type}_${start}_${end}**. By default, two domains with identical types, start and end positions, are considered overlapping and will trigger an exception, but if the types are different, no such issue occurs. This is to avoid a scenario where multiple redundant domains are added, although if this is desired, this can be overridden using the **autoname** argument when adding domains. SHEPHARD also provides functionality to automatically construct domains based on Tracks, enabling the automatic discretization of continuous data into defined subregions. Domains are appropriate for structural information (e.g., annotated folded domains, helices, *etc.*), defined functional domains, or shorter sequence motifs.

***Site objects*** map to a single position within a protein sequence. A Site is defined by a position, a site type, the associated protein, and symbolic or numerical data. Multiple sites can coexist at the same residue. Sites are appropriate for specific positional information such as single point mutations, post-translational modifications, or known binding residues.

For Domains, information from Tracks or Sites found within the domain can be directly extracted. Similarly, for Sites, Track-specific data can be obtained, as can Domains that encompass a Site. We can interrogate both Proteomes and Proteins for Domains or Sites of a specific type, and we can identify proteins from which Sites of a specific type originate. In effect, the SHEPHARD data structure offers a relational database grounded using a Primary Key (**protein.unique_ID**) associated with each protein, yet offering syntactically intuitive ways to query and analyze in what feels like conventional Python code.

***Generating SHEPHARD Proteomes***

Generating and populating proteomes into SHEPHARD can be achieved in several ways.

The recommended route is to start with a FASTA file with one or more sequence records (**Fig. S6**). The FASTA format stipulates that each sequence is demarked with a header line that starts with a caret (>) character followed by identifying information. FASTA files can be used to create new Proteomes using the FASTA API (**shephard.apis.fasta**).

from shephard.apis import fasta

# will assign each protein a unique numerical index starting with 1

local_proteome = fasta.fasta_to_proteome(‘sequence_file.fasta’)

# will use the FASTA header as a unique ID

local_proteome = fasta.fasta_to_proteome(‘sequence_file.fasta’, use_header_as_unique_ID=True)

In addition, if the FASTA file was obtained from UniProt (<http://uniprot.org/>), then the UniProt API (**shephard.apis.uniprot**) can be used to parse a FASTA file such that UniProt accession is used as the protein unique ID and the protein name is parsed from the FASTA header.

from shephard.apis import uniprot

# will assign each protein a unique numerical index starting with 1

local_uniprot_proteome = uniprot.uniprot_fasta_to_proteome(‘sequence_file.fasta’)

In addition to loading data from FASTA files, SHEPHARD enables previously-generated SHEPHARD protein files to be read using the proteins SHEPHARD interface (**shephard.interfaces.si_proteins**). SHEPHARD-formatted protein files include a single line per protein, as described in the reference implementation for the proteins files (defined in the online documentation <https://shephard.readthedocs.io/en/latest/shephard_file_types.html>). As with FASTA files, these files can be read to generate a new Proteome object in a single line of code.

from shephard import Proteome

# create an empty Proteome object

P = Proteome()

# annotate the Proteome with proteins

si_proteins.add_proteins_from_file(P, 'shprd_proteins_file.tsv')

Finally, proteomes can be constructed programmatically. A new Proteome object can be created using an empty constructor, and then proteins can be added using the (**add_protein()**) function. New proteins require a sequence, a unique ID, and a name and can have zero or more attributes.

from shephard import Proteome

# create an empty Proteome object

P = Proteome()

# Add a new protein with an attribute

P.add_protein('PREWTEINSEQWENCE', 'demo protein', 'XXA', attributes={'source':'this protein was added directly in code'})

print(len(P))

> 1

***Annotating proteins programmatically***

Proteins can be annotated directly from Python to add new features, which can then be exported and import in the future. Annotations can be Protein attributes, Sites, Domains, or Tracks, as shown below

from shephard import Proteome

# create an empty Proteome object

P = Proteome()

# Add a new protein with an attribute

P.add_protein('PREWTEINSEQWENCE', 'demo protein', 'idx_1')

# excise the new Protein from the Proteome object

local_protein = P.protein('idx_1')

# annotate the Protein with an attribute

local_protein.add_attribute('date',2023)

# annotate the Protein with a Site - in this case:

# site_type = ‘phosphosite’

# symbol = ‘T’

# value = 100

local_protein.add_site(5, 'phosphosite', symbol='T', value=100)

# annotate the Protein with a Domain (the domain here is between residues 1 and 3)

local_protein.add_domain(1,3, 'NTD')

# annotate the Protein with a Track (here the track is symbol the symbol ‘X’ assigned to

# every position, but tracks can also be numerical (in which case we’d assign values=<?>

# instead of here where we do symbols=<?>

local_protein.add_track('all_X', symbols=['X']*len(local_protein))

In this way, complex analysis pipelines can be developed whereby Proteins are bioinformatically annotated, and those annotations can then be expired (as described below) for ease of use in future analyses.

Of note, Domains, and Sites can also be annotated with attributes in exactly the same way.

***Importing SHEPHARD protein annotations***

Once a Proteome has been generated, it can be annotated with Tracks, Domains, Sites, or Attributes. In all four cases, these annotations can be done using functions from the appropriate interface model in (**shephard.interfaces**). Interfaces implement consistent, stateless functions that enable the reading (and writing) of SHEPHARD data from (and to) a standardized file format. This file format SHEPHARD uses by default is a simple tab-separated text format that is easily generated or readable using commonly used software (e.g., Excel). The objective is to make it easy to read (and write) data into (and out of) SHEPHARD.

By way of example, for annotating a Proteome object with domains, one would use the **shephard.interfaces.si_domains.add_domains_from_file()** function. This function takes two arguments: a Proteome object and the path to the Domains file. During parsing of the Domains file, SHEPHARD ensures that annotations do not extend beyond the positions available in a domain, and only annotations for Protein objects found in the Proteome are added.

In addition to reading annotations from an annotation file, **shephard.interfaces** enable dictionaries of annotations to be used to annotate proteomes programmatically. The specifics for dictionary-derived annotations are available in the SHEPHARD reference documentation. The main advantage here is that using a dictionary-based annotation enables dynamic annotations to be added, allowing SHEPHARD to be integrated into an existing software pipeline without the need for writing to disk.

***Exporting SHEPHARD protein sequence information***

As with reading protein sequence information, writing sequence information can be achieved in two different ways. Proteome objects can be exported to FASTA file via the FASTA API (**api.fasta**).

***Exporting SHEPHARD annotations***

Annotations from a Proteome object can be exported for future use or distribution. There are two ways this can be done. In one approach, the Proteome object is passed into one of the **write_<annotation>** functions (e.g., **shephard.interfaces.si_sites.write_sites()**). These functions take a Proteome object, an output path, and then enable specific types of annotations to be written based on (for example) Site type or Domain Type.

The alternative approach is to manually generate a Python list of the annotations of interest and then pass this list of annotation objects to a **write_<x>_from_list** function. For example, to write a list of sites the function **shephard.interfaces.si_sites.write_sites_from_list()** may be used. This approach provides much finer-grain control over which annotations are actually written - for example, one could iterate through all the sites in a Proteome and add only those from proteins under some threshold number of residues to a new list (using standard Python list syntax of **append()**).

These two modes of data export enable the two standard scenarios: either a user wants to export all the annotations of a specific type or class to facilitate full reproducibility, or a user wants to export a very specific set of annotations for further analysis downstream.

***SHEPHARD Application Programming Interfaces (APIs)***

While SHEPHARD guarantees the ability to read and write in its standardized formats, some additional formats are sufficiently broadly used that they merit their own specific APIs for reading and writing. As introduced above, FASTA files are the *de facto* standard format for sharing protein sequence information, and, as such, ensuring SHEPHARD provides robust capabilities for reading and writing FASTA files is important.

For standard FASTA file reading/writing, the functions encapsulated by the fasta module should be sufficient (e.g., **shephard.apis.fasta**). In addition, given we generally recommend working with data obtained from UniProt for proteome-wide analysis, we provided an UniProt API for reading/writing FASTA data obtained from UniProt (e.g., **shephard.apis.uniprot**). More broadly, we plan to add additional APIs for specific tools or packages as soft dependencies moving forward; i.e. APIs provide a means for SHEPHARD to include modular gateways to additional functionality. Moreover, APIs are not a core component of SHEPHARD and instead should be thought of as a convenient layer of regulation and functionality.

***SHEPHARD tools***

In addition to core functionality encoded within different SHEPHARD objects, we provide a set of stand-alone tools for manipulating SHEPHARD data. These modules include **shephard.tools.domain_tools**, **shephard.tools.sequence_tools**, **shephard.tools.site_tools** and **shephard.tools.track_tools**.

The tools modules share the fact that all functions are stateless, stand-alone, and do not modify the objects passed into them. In this way, tool functions enable the manipulation or analysis of existing data. SHEPHARD annotation objects make use of functions encoded in the tools modules, but in addition, certain functionality that may of use or of interest for those using SHEPHARD are encoded in the tools modules

In particular, the **shephard.tools.track_tools.build_track_from_domains()** function enables a set of domains to be converted into a track. The corollary is also possible with the **shephard.tools.domain_tools.build_domains_from_track()** function. This is particularly useful for identifying regions that can discretize a continuous (track) annotation into a binary (domain) annotation. For example, one could use this to identify ‘domains’ with a high propensity for some predictable value.

While the **tools** modules contain relatively few functions at the time of writing, our goal is to have these modules as a public-facing location for additional functions that may emerge. In this way, pull requests for new types of features can be implemented here in a way that, by definition, does not impact the core components of the SHEPHARD architecture. In much the same way that the APIs are designed to be modular and extensible, so too are the **tools** modules.

***INSTALLING AND USING SHEPHARD***

SHEPHARD can be installed from the Python packaging index using the package installer for Python (**pip**) tool. Specifically, when run from the terminal, installation can be achieved using the following command:

**pip install shephard**

Documentation for using SHEPHARD is available at <https://shephard.readthedocs.io/>.

In addition to a collection of example notebooks for SHEPHARD, we provide a set of Google-colab notebooks which are linked from <https://github.com/holehouse-lab/shephard-colab>. These notebooks are provided to illustrate interactive examples of the types of analysis SHEPHARD can provide.

***SHEPHARD’S “VALUE ADDED”***

SHEPHARD was designed to simplify the mundane but absolutely essential components of data sanitizing, error checking, and organization when working with large protein datasets. While we have found it to dramatically improve our ability to design and execute large-scale bioinformatics pipelines, the “value added” is difficult to formally assess, as it depends entirely on the problem being addressed and the individual. For example, SHEPHARD hides and automates many of the I/O and data cleaning steps that should be done when working with heterogeneous data, yet for an inexperienced bioinformatician best practices and standard design patterns for these components of analysis may be unfamiliar, meaning SHEPHARD could save days to weeks. In contrast, for a seasoned bioinformatician, it may only save an hour or so. Nevertheless, in our own experience we have found the ability to rapidly proptype ideas catalyzes new questions and lubricates the execution of so-called “night science”^4^.

The entire point of SHEPHARD as a framework is that it levels the playing field for novice bioinformaticians while simultaneously providing a set of tools that makes performing reliable and reproducible analysis straightforward. For “power users”, SHEPHARD automates big chunks of what would either need to be written from scratch or co-opted from prior projects, making it simple to develop and deploy complex bioinformatic pipelines.

SHEPHARD does not enable analyses that could not have been done previously. However, it does make it trivial to perform analyses that currently necessitate a series of steps that are insurmountably high for many (most) scientists.

Beyond simply providing this package, we have precomputed a large set of sequence features and shared these precomputed analyses as a Google colab notebook, as well as the raw data via our SHEPHARD data distribution portal (<https://github.com/holehouse-lab/shephard-data>). With this in mind, someone with just a basic understanding of the Python programming language and a web-browser can perform Proteome-wide analysis of the human proteome.

From our own experience, the steps that would be necessary to analyze UniProt annotations without SHEPHARD are as follows.

1. Gain familiarity with conda, pip, and Python environment management, as well as sufficient expertise in Python to complete steps 3 – 6.
2. Become familiar with UniProt in terms of the types of annotations, how they are arranged and organized etc.
3. Learn how to use the **requests** package to make an HTTP call to the UniProt API, including writing RESTFUL API calls. Alternatively, this could be done using **wget** or **curl** from the command-line, and the resulting data parsed from a file. It is unclear which of these would be the “easier” option, as ease here is entirely subjective.
4. Learn how to parse the resulting XML file. Specifically, this involves:
   1. Following the UniProt API XML schema (requests returns XML as a collection of dictionaries and lists which can only be understood if the underlying scema is referenced).
   2. Parse out the annotations of interest into an appropriate data structure.
   3. Do so in a manner that is robust to exceptions and sanity checks during data acquisition to ensure only valid annotations are obtained.
5. Write functions to cross reference annotations from XML-parsed annotation data to amino acid sequence, ensuring that Python indexing (from 0) and biological indexing (from 1) are appropriately offset.
6. Writing code to iteratively analyze annotations in the relevant sequence context, including (ideally) sanity checking and defensive programming to minimize errors.

In contrast, SHEPHARD (using preannotations) enables the analyses to be performed out the box.

2. DATA USED IN THIS STUDY

All the data and associated analysis scripts described below are provided in the main GitHub repository at:

<https://github.com/holehouse-lab/supportingdata/tree/master/2022/ginell_2022>

For each of these datasets, a specific discussion is provided below.

***Human proteome***

The human proteome was obtained from UniProt in 2020 and used as a reference for this study ^5^. In part, this helps maintain consistency with the AlphaFold2 data, which was obtained in July 2021 from the initial (V1) AlphaFold/EBI data deposition ^6,7^. Note that we compared the predicted structures for the human proteome with the second AlphaFold2/EBI data deposition (December 2021) and found almost no changes - a full description of this is available under the **/misc** directory on the SHEPHARD manuscript GitHub page

To ensure compatibility with the provided AlphaFold2 data, any proteins without an exact sequence match between the AlphaFold2 data and the model human proteome were filtered out. This left 20,061 proteins (98.4% of the original human proteome). This ultimately left 326,924 PTM annotations (99.8% of the original annotated PTMs). It is worth noting that the filtering here has no material impact on the results.

All Proteomes were similarly obtained from the UniProt Proteomes interface found at <https://www.uniprot.org/proteomes>.

Proteins with invalid amino acids were removed using protfasta ^3^ using the pfasta command-line tool. As an example:

**pfasta --invalid-sequence remove raw_human_proteome.fasta -o human_proteome_validated.fasta**

***Intrinsically disordered domains***

Intrinsically disordered regions were predicted using metapredict V2 ^8,9^.

Specifically, we used the command-line tool **metapredict-predict-idrs** with the **--mode** set to **shephard-domains** flag.

**metapredict-predict-idrs human_proteome_validated.fasta --mode shephard-domains-uniprot \\**

**-o shprd_domains_metapredictv2.tsv --verbose**

We note that proteome-wide disorder predictions can also be made using the metapredict Google colab notebook linked from <https://metapredict.net/>.

***Polar-rich low complexity domains (pLCDs)***

Polar-rich low complexity domains (pLCDs) were identified using the method developed by Gutierrez *et al.* ^10^. Specifically, this approach identifies contiguous regions enriched in specific residues, enabling gap sizes and minimum region sizes to be defined. We selected regions in the human proteome rich in polar and proline residues (Q, S, G, N, T, & P), with a maximum interruption of 5 residues, a minimum domain size of 50 residues, and a fractional threshold of 0.5 or higher. Code for computing this is provided at the GitHub repository under **/misc/find_plcds/**, and the specific implementation is provided in the sequence analysis package, sparrow (<https://github.com/idptools/sparrow>).

For pLCDs, we then selected only LCDs that overlap with IDRs, and calculated the fractions of polar, aromatic, aliphatic, and charge residues in each region. Chemically distinct polar LCDs are then defined as LCDs that are depleted in two of the chemical types but above a specific relative threshold in the chemical type of interest. In our specific analysis, the thresholds used were (1) in the bottom 5% of the cumulative distribution for the combined two depletion chemistries and (2) in the top 20% for the chemistry of interest. For example, charge-rich polar low complexity domains were those domains that were in the bottom 5% for aromatic and aliphatic content, but the top 20% for charge. Depletion thresholds were determined using a cumulative distribution function and selecting a threshold at the X-percentile of the entire proteome. The depletion threshold implemented depends directly on the question of interest. In the example analysis, the depletion threshold is determined based on the proteome in question, however a threshold could alternatively be selected to match a predefined mean fraction of residues. Ultimately we see this method for PLDs, as a convenient, reproducible mechanism to identify and select chemically analogous IDRs.

In the spirit of completeness, we also calculated a large collection of chemically distinct LCDs, all of which are provided as a SHEPHARD domains file under **/misc/find_plcds/ shprd_domains_human_LCDs_all.tsv**.

**Post-translational modifications (PTM)**

PTM data were obtained from ProteomeScout (downloaded summer of 2022) and parsed using the ProteomeScoutAPI ^11,12^. Specifically, PTM data were downloaded from <https://proteomescout.wustl.edu/compendia>.

**Structural annotations**

Structural annotations across the human proteome were based on predicted protein structures from AlphaFold2 ^6,7^. The decision to use computed structural models for proteome-wide structural analysis reflects the convenience this affords with respect to guaranteed coverage, 1:1 mapping between sequence and structure, and the remarkable accuracy that AlphaFold2 offers. Classification into distinct structural subclasses was performed using the DISICL classification scheme developed by Nagy *et al.* and implemented in the SESCA package ^13,14^. We also employed the DSSP algorithm to annotate the human proteome based on the AlphaFold2 annotations ^15^.

**Accessible residues**

Residue accessibility was calculated using SOURSOP (<https://soursop.readthedocs.io/>) and MDTraj ^16^, using a solvent probe radius of 7 Å. Code for computing this is provided at the GitHub repository under **/PTM_af2_analysis/residue_accessibility/**.

**Gene ontology enrichment**

Gene ontology (GO) enrichment was performed using PANTHER ^17^. For arginine and lysine-rich IDR proteins, we enriched was calculated by comparing UniProt IDs for proteins identified in our analysis against the entire human proteome (Table S3, S4). For polar-rich low-complexity domains, we calculated enrichment compared to both the complete human proteome (using PANTHER Overrepresentation Test - Released 20220712) (Table S5, S6, S7) and all IDR-containing proteins (using PANTHER Overrepresentation Test - Released 20221013) (Table S8, S9, S10). In both cases, identical trends were observed, and almost identical sets of GO terms were found (compare tables S5 to S10).

**Protein abundance data**

Protein abundance data were included for humans ^18^, *A. thaliana* ^19^, *D. melanogaster* ^20^, *E. coli* ^21,22^, *S. pombe* ^23^, and *S. cerevisiae ^24,25^*. To calculate correlations, we took the set of proteins for which abundance data were available and separated them into ventiles (twenty equally-spaced groups, rank-ordered from lowest to highest expression levels). The use of ventiles was primarily to ensure we also had groups with sufficiently large numbers of proteins, although where accessible changing the number of bins did not alter the results. For each ventile, we calculated the average fraction disorder of proteins, the average number of disordered residues, and the average fraction of charged residues (FCR) for disordered regions. The correlation between ventiles and the calculated property was then computed using Pearson’s correlation coefficient. Code for computing this is provided in the GitHub repository under **IDR_copy_number/copy_number_analysis.ipynb**.

**Prion-like domains**

Prion-like domains were predicted using the PLAAC algorithm ^26,27^. The PLAAC source code was obtained from <https://github.com/whitehead/plaac>, and run using default parameters to analyze the human proteome.

3. SUPPLEMENTARY TABLES

|  | Number of residues | Number of PTM sites | Percentage of residues that are modified |
| --- | --- | --- | --- |
| Human proteome | 10,483,347 | 312,745 | 3.0 % |
| Human IDRs | 3,439,190 | 154,758 | 4.5 % |

**Table S1.** IDRs are disproportionately post-translationally modified. On average, 4.5% of all disordered residues have one or more post-translational modifications. Moreover, 50% of all residues that have one or more modifications are found in IDRs, even though only 33% of residues in the human proteome are found in IDRs. Note that PTM sites are defined as residues with one or more post-translational modifications; i.e., a residue that received two different types of modifications would be counted once.

| **Post-translational modification** | **Count** |
| --- | --- |
| Phosphoserine | 131,681 |
| Phosphothreonine | 54,168 |
| Phosphotyrosine | 38,412 |
| Ubiquitination | 38,196 |
| N6-acetyllysine | 20,331 |
| N-Glycosylation | 13,882 |
| Methylation | 11,535 |
| Sumoylation | 6,528 |
| Dimethylation | 1,375 |
| O-Glycosylation | 1,255 |
| Omega-N-methylarginine | 1,154 |
| N6-succinyllysine | 1,152 |
| N-acetylalanine | 949 |
| N-acetylmethionine | 820 |
| S-nitrosocysteine | 729 |
| N6,N6-dimethyllysine | 504 |
| Asymmetric dimethylarginine | 477 |

**Table S2.** PTMs of interest across the human proteome. We focus on the most numerous 17 types of modifications.

| **Term** | **Total** | **Actual** | **Expected** | **Enrich.** | **Raw P value** | **FDR** |
| --- | --- | --- | --- | --- | --- | --- |
| regulation of RNA splicing (GO:0043484) | 75 | 37 | 4.28 | 8.64 | 8.47E-20 | 2.35E-17 |
| mRNA splicing, via spliceosome (GO:0000398) | 176 | 66 | 10.05 | 6.57 | 9.32E-29 | 5.18E-26 |
| RNA splicing, via transesterification reactions with bulged adenosine as nucleophile (GO:0000377) | 176 | 66 | 10.05 | 6.57 | 9.32E-29 | 4.14E-26 |
| RNA splicing, via transesterification reactions (GO:0000375) | 176 | 66 | 10.05 | 6.57 | 9.32E-29 | 3.45E-26 |
| RNA splicing (GO:0008380) | 221 | 77 | 12.62 | 6.1 | 1.23E-31 | 1.37E-28 |
| mRNA processing (GO:0006397) | 229 | 79 | 13.08 | 6.04 | 3.41E-32 | 7.58E-29 |
| regulation of mRNA metabolic process (GO:1903311) | 110 | 32 | 6.28 | 5.09 | 3.78E-12 | 5.25E-10 |
| mRNA metabolic process (GO:0016071) | 316 | 88 | 18.05 | 4.88 | 5.18E-30 | 3.84E-27 |
| calcium ion transmembrane transport (GO:0070588) | 80 | 19 | 4.57 | 4.16 | 1.25E-06 | 6.93E-05 |

**Table S3. GO enrichment terms for proteins with arginine-rich IDRs.** Proteins with arginine-rich IDRs are enriched for RNA-associated annotations, along with the curious inclusion of calcium ion transmembrane transport.

| **Term** | **Total** | **Actual** | **Expected** | **Enrich.** | **Raw P value** | **FDR** |
| --- | --- | --- | --- | --- | --- | --- |
| chromatin remodeling (GO:0006338) | 77 | 30 | 3.84 | 7.81 | 1.26E-15 | 2.00E-13 |
| chromatin organization (GO:0006325) | 118 | 35 | 5.89 | 5.95 | 5.24E-15 | 7.75E-13 |
| DNA conformation change (GO:0071103) | 74 | 20 | 3.69 | 5.42 | 1.36E-08 | 5.30E-07 |
| DNA recombination (GO:0006310) | 115 | 26 | 5.74 | 4.53 | 2.48E-09 | 1.06E-07 |
| protein-DNA complex subunit organization (GO:0071824) | 83 | 18 | 4.14 | 4.35 | 1.17E-06 | 3.16E-05 |
| chromosome organization (GO:0051276) | 256 | 53 | 12.77 | 4.15 | 3.36E-16 | 5.73E-14 |

**Table S4. GO enrichment terms for proteins with lysine-rich IDRs**. Proteins with lysine-rich IDRs are enriched for DNA-associated annotations (notably those associated with chromatin and chromosomal annotations).

|  | **Proteins with aromatic-rich pLCDs** | | | | | | |
| --- | --- | --- | --- | --- | --- | --- | --- |
|  | **Term** | **Total** | **Actual** | **Expected** | **Enrich.** | **Raw P value** | **FDR** |
| **Cellular Component** | keratin filament (GO:0045095) | 31 | 4 | 0.13 | 30.54 | 1.39E-05 | 3.54E-03 |
|  | nuclear pore (GO:0005643) | 47 | 4 | 0.2 | 20.14 | 6.28E-05 | 1.07E-02 |
|  | intermediate filament (GO:0005882) | 50 | 4 | 0.21 | 18.93 | 7.88E-05 | 1.01E-02 |
|  | intermediate filament cytoskeleton (GO:0045111) | 51 | 4 | 0.22 | 18.56 | 8.47E-05 | 8.65E-03 |
| **Molecular Function** | structural constituent of nuclear pore (GO:0017056) | 19 | 4 | 0.08 | 49.82 | 2.44E-06 | 4.43E-04 |
|  | single-stranded RNA binding (GO:0003727) | 33 | 3 | 0.14 | 21.51 | 4.65E-04 | 3.62E-02 |
|  | signal sequence binding (GO:0005048) | 33 | 3 | 0.14 | 21.51 | 4.65E-04 | 3.17E-02 |
|  | RNA binding (GO:0003723) | 617 | 20 | 2.61 | 7.67 | 1.52E-12 | 8.33E-10 |
|  | structural molecule activity (GO:0005198) | 230 | 7 | 0.97 | 7.2 | 6.38E-05 | 5.81E-03 |

**Table S5. GO enrichment terms for proteins with aromatic-rich polar-rich low-complexity domains (aromatic-rich pLCDs)**. Proteins identified include those associated with RNA binding, as well as those with structural roles in subcellular organization. Enrichment for these results was calculated against the entire human proteome.

|  | **Proteins with charge-rich pLCDs** | | | | | | |
| --- | --- | --- | --- | --- | --- | --- | --- |
|  | **Term** | **Total** | **Actual** | **Expected** | **Enrich.** | **Raw P value** | **FDR** |
| **Biological process** | mRNA splicing, via spliceosome (GO:0000398) | 176 | 9 | 0.9 | 10.03 | 4.31E-07 | 2.39E-04 |
|  | RNA splicing, via transesterification reactions (GO:0000375) | 176 | 9 | 0.9 | 10.03 | 4.31E-07 | 1.60E-04 |
|  | mRNA processing (GO:0006397) | 229 | 11 | 1.17 | 9.42 | 3.88E-08 | 8.61E-05 |
|  | RNA splicing (GO:0008380) | 221 | 10 | 1.13 | 8.87 | 2.83E-07 | 2.09E-04 |
|  | mRNA metabolic process (GO:0016071) | 316 | 11 | 1.61 | 6.83 | 8.62E-07 | 2.73E-04 |
|  | RNA processing (GO:0006396) | 481 | 12 | 2.45 | 4.89 | 7.67E-06 | 1.55E-03 |
| **Cellular component** | transcription elongation factor complex (GO:0008023) | 34 | 5 | 0.17 | 28.84 | 1.53E-06 | 1.31E-04 |
|  | nuclear body (GO:0016604) | 50 | 6 | 0.25 | 23.53 | 3.90E-07 | 6.65E-05 |
|  | nucleoplasm (GO:0005654) | 422 | 15 | 2.15 | 6.97 | 5.73E-09 | 1.46E-06 |
|  | nuclear lumen (GO:0031981) | 632 | 15 | 3.22 | 4.65 | 9.35E-07 | 9.55E-05 |
|  | membrane-enclosed lumen (GO:0031974) | 731 | 15 | 3.73 | 4.02 | 5.35E-06 | 3.91E-04 |
|  | intracellular organelle lumen (GO:0070013) | 731 | 15 | 3.73 | 4.02 | 5.35E-06 | 3.42E-04 |
|  | organelle lumen (GO:0043233) | 731 | 15 | 3.73 | 4.02 | 5.35E-06 | 3.04E-04 |
| **Molecular function** | transcription coregulator activity (GO:0003712) | 210 | 7 | 1.07 | 6.54 | 1.21E-04 | 2.20E-02 |
|  | RNA binding (GO:0003723) | 617 | 16 | 3.15 | 5.08 | 1.22E-07 | 6.66E-05 |

**Table S6. GO enrichment terms for proteins with charge-rich polar-rich low-complexity domains (charge-rich pLCDs)**. Proteins identified include many associated with nuclear function and nuclear subcellular localization. These include RNA processing, splicing, and proteins associated with the nucleoplasm. Enrichment for these results was calculated against the entire human proteome.

|  | **Proteins with aliphatic-rich pLCDs** | | | | | | |
| --- | --- | --- | --- | --- | --- | --- | --- |
|  | **Term** | **Total** | **Actual** | **Expected** | **Enrich.** | **Raw P value** | **FDR** |
| **Biological Process** | transcription by RNA polymerase II (GO:0006366) | 1438 | 51 | 8.17 | 6.24 | 9.80E-28 | 5.44E-25 |
|  | regulation of transcription by RNA polymerase II (GO:0006357) | 1383 | 47 | 7.86 | 5.98 | 1.52E-24 | 1.77E-22 |
|  | nucleic acid-templated transcription (GO:0097659) | 1790 | 58 | 10.17 | 5.7 | 3.79E-30 | 8.42E-27 |
|  | RNA biosynthetic process (GO:0032774) | 1798 | 58 | 10.22 | 5.68 | 4.78E-30 | 3.53E-27 |
|  | regulation of transcription, DNA-templated (GO:0006355) | 1713 | 53 | 9.73 | 5.44 | 3.37E-26 | 6.23E-24 |
|  | regulation of RNA metabolic process (GO:0051252) | 1853 | 56 | 10.53 | 5.32 | 1.98E-27 | 8.81E-25 |
|  | regulation of nucleobase-containing compound metabolic process (GO:0019219) | 1911 | 56 | 10.86 | 5.16 | 9.03E-27 | 2.23E-24 |
|  | regulation of cellular macromolecule biosynthetic process (GO:2000112) | 1849 | 53 | 10.51 | 5.04 | 1.16E-24 | 1.61E-22 |
|  | nucleobase-containing compound biosynthetic process (GO:0034654) | 2026 | 58 | 11.51 | 5.04 | 2.16E-27 | 8.00E-25 |
|  | regulation of gene expression (GO:0010468) | 2106 | 57 | 11.97 | 4.76 | 1.28E-25 | 1.90E-23 |
|  | RNA metabolic process (GO:0016070) | 2410 | 61 | 13.7 | 4.45 | 2.96E-26 | 5.97E-24 |
| **Molecular function** | transcription regulator activity (GO:0140110) | 1265 | 39 | 7.19 | 5.43 | 1.22E-18 | 3.32E-16 |
|  | RNA polymerase II transcription regulatory region sequence-specific DNA binding (GO:0000977) | 1059 | 32 | 6.02 | 5.32 | 5.69E-15 | 4.44E-13 |
|  | DNA binding (GO:0003677) | 1361 | 41 | 7.73 | 5.3 | 2.65E-19 | 1.44E-16 |
|  | nucleic acid binding (GO:0003676) | 1974 | 46 | 11.22 | 4.1 | 1.60E-17 | 2.92E-15 |

**Table S7. GO enrichment terms for proteins with aliphatic-rich polar-rich low-complexity domains (aliphatic-rich pLCDs)**. Proteins identified include mostly those involved in transcriptional regulation across a range different processes, including RNA synthesis. Enrichment for these results was calculated against the entire human proteome.


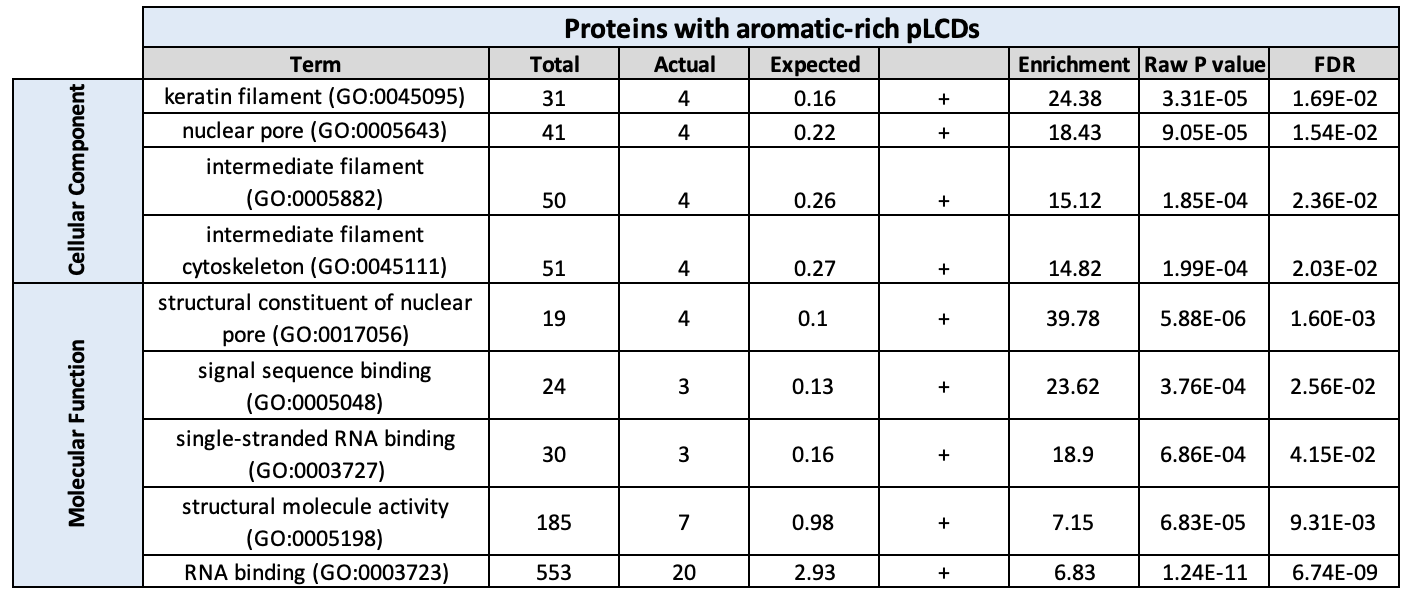


**Table S8. GO enrichment terms for proteins with aromatic-rich polar-rich low-complexity domains (aromatic-rich pLCDs)**. Proteins identified include those associated with RNA binding, as well as those with structural roles in subcellular organization. Enrichment for these results was calculated against IDR-containing proteins only (n = 16,439).


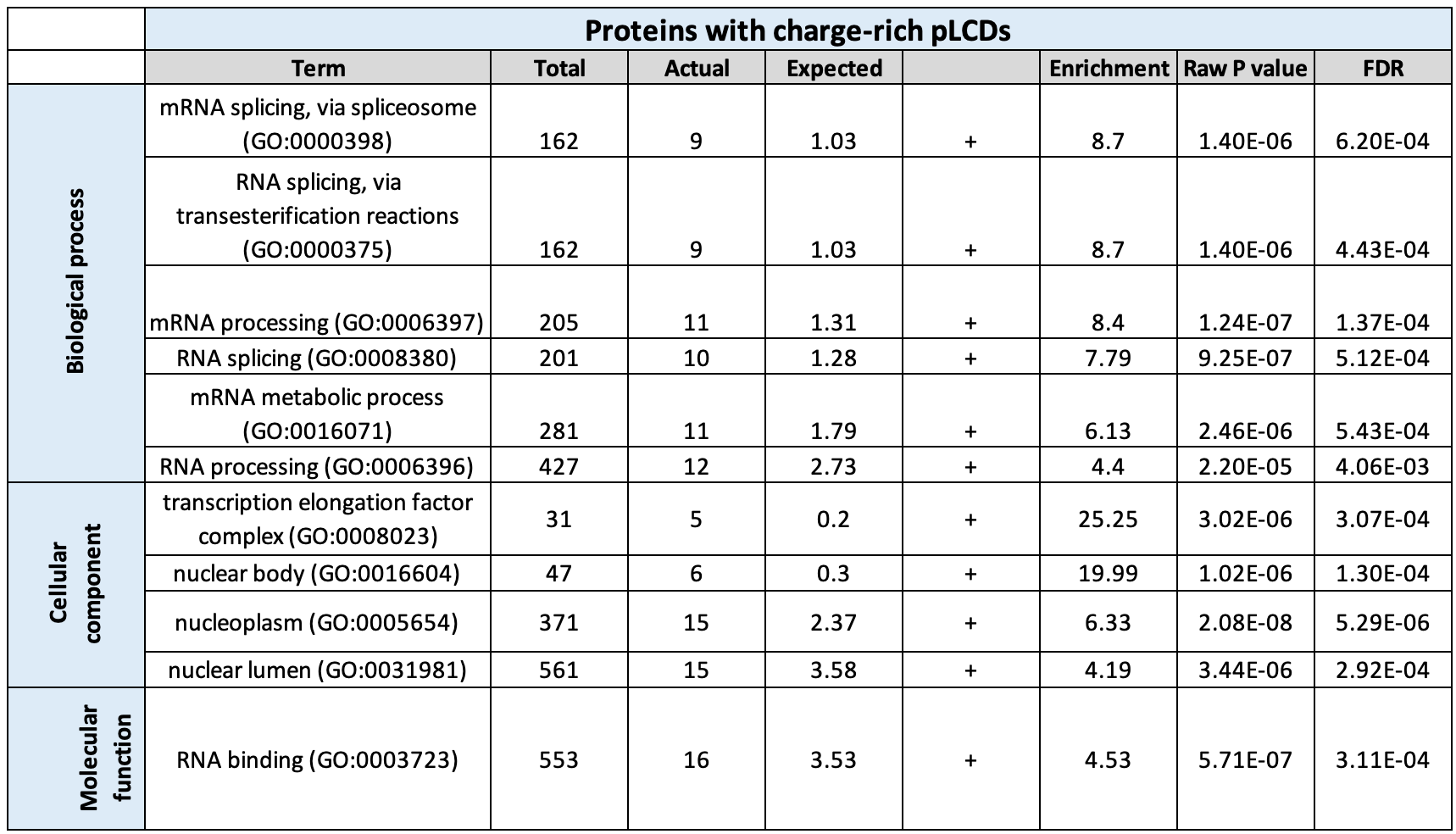


**Table S9. GO enrichment terms for proteins with charge-rich polar-rich low-complexity domains (charge-rich pLCDs)**. Proteins identified include many associated with nuclear function and nuclear subcellular localization. These include RNA processing, splicing, and proteins associated with the nucleoplasm. Enrichment for these results was calculated against IDR-containing proteins only (n = 16,439).


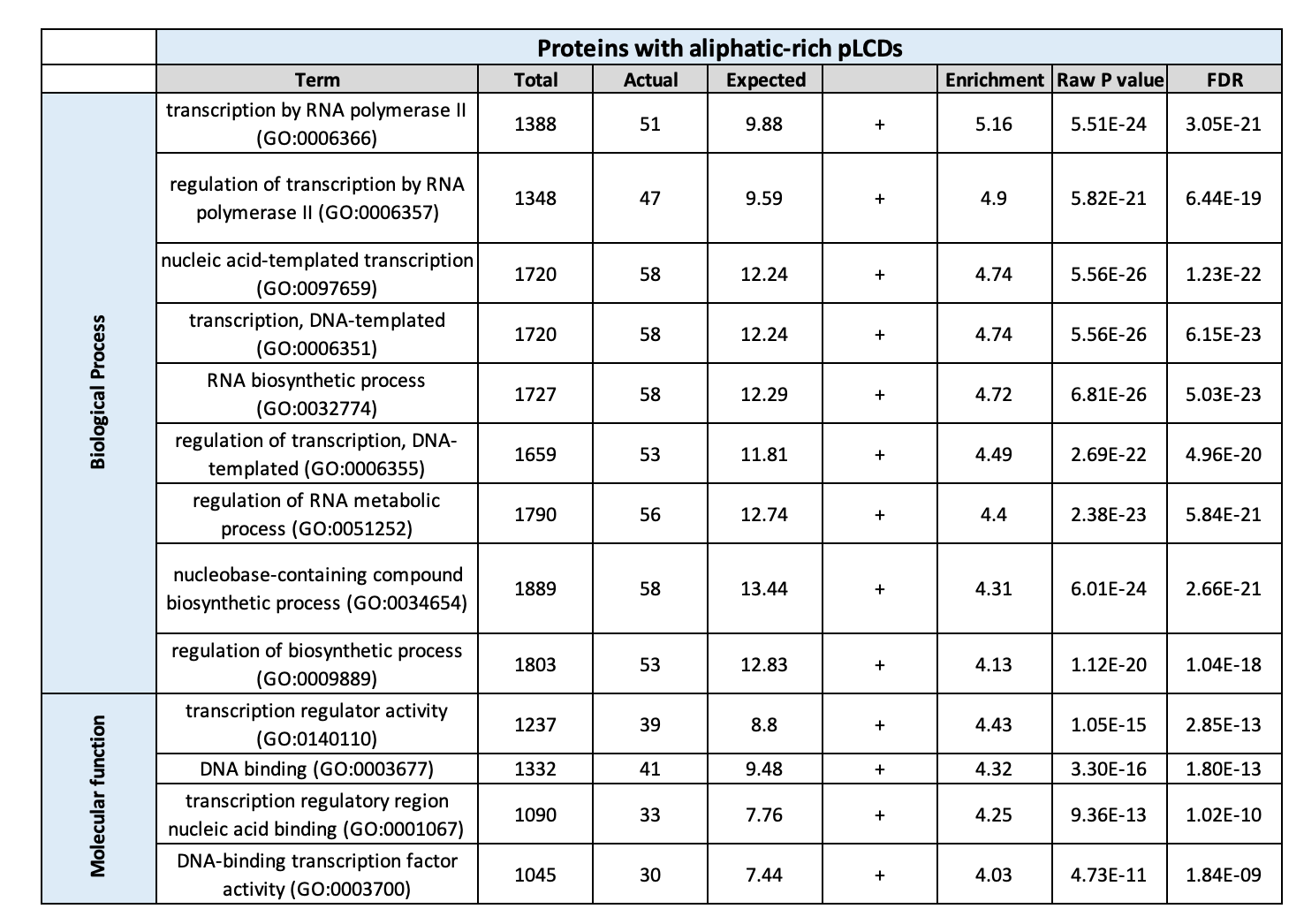


**Table S10. GO enrichment terms for proteins with aliphatic-rich polar-rich low-complexity domains (aliphatic-rich pLCDs)**. Proteins identified include mostly those involved in transcriptional regulation across a range different processes, including RNA synthesis. Enrichment for these results was calculated against IDR-containing proteins only (n = 16,439).

4. SUPPLEMENTARY FIGURES

**
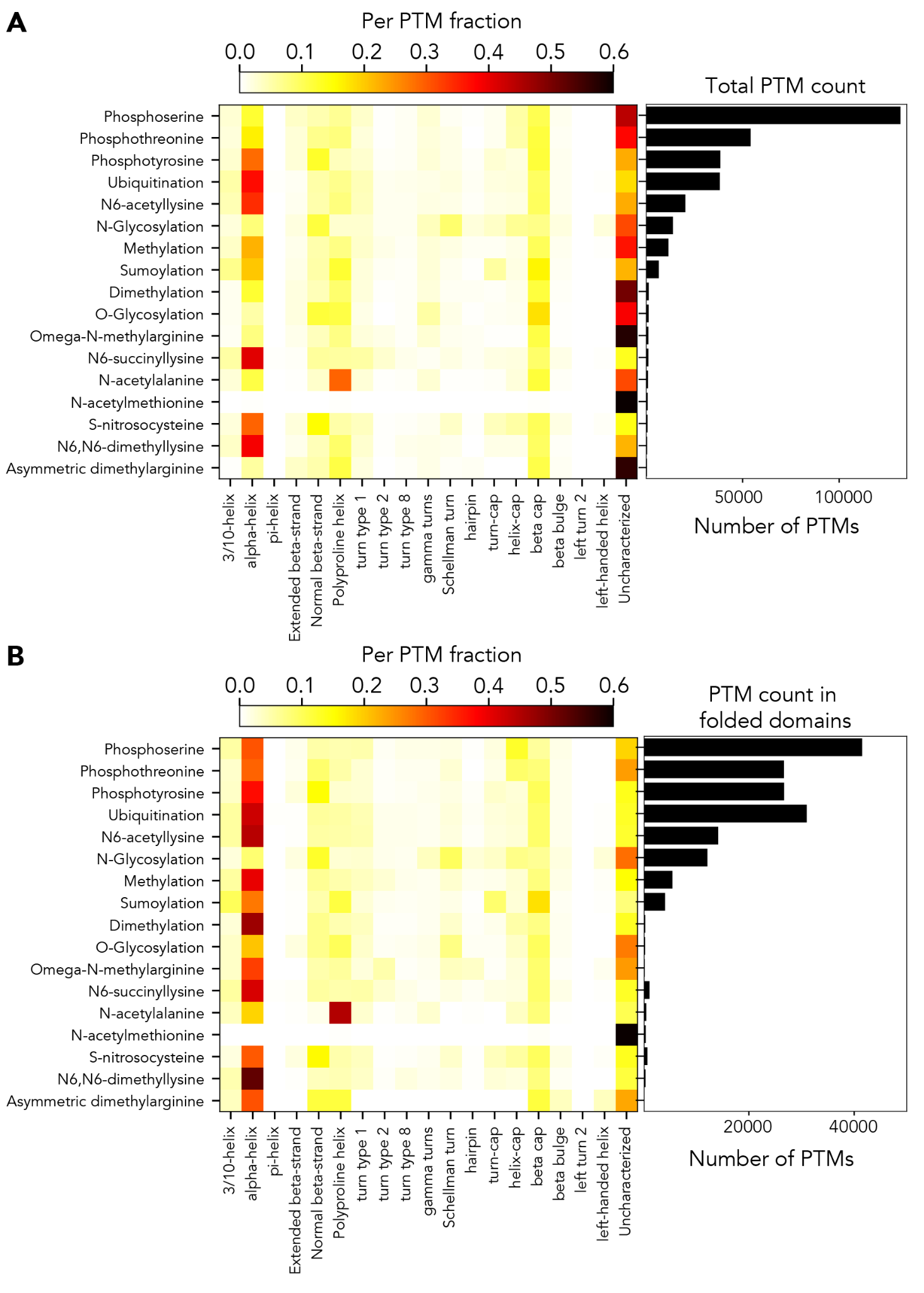
**

**Figure S1. PTMs show strong structural biases for the local structural context.** **(A)** PTMs overwhelmingly fall in regions that lack a defined secondary structure or alpha helices. **(B)** If only PTMs in folded regions are analyzed, similar trends are recovered, with the balance between short disordered loops and alpha helices largely swapping.


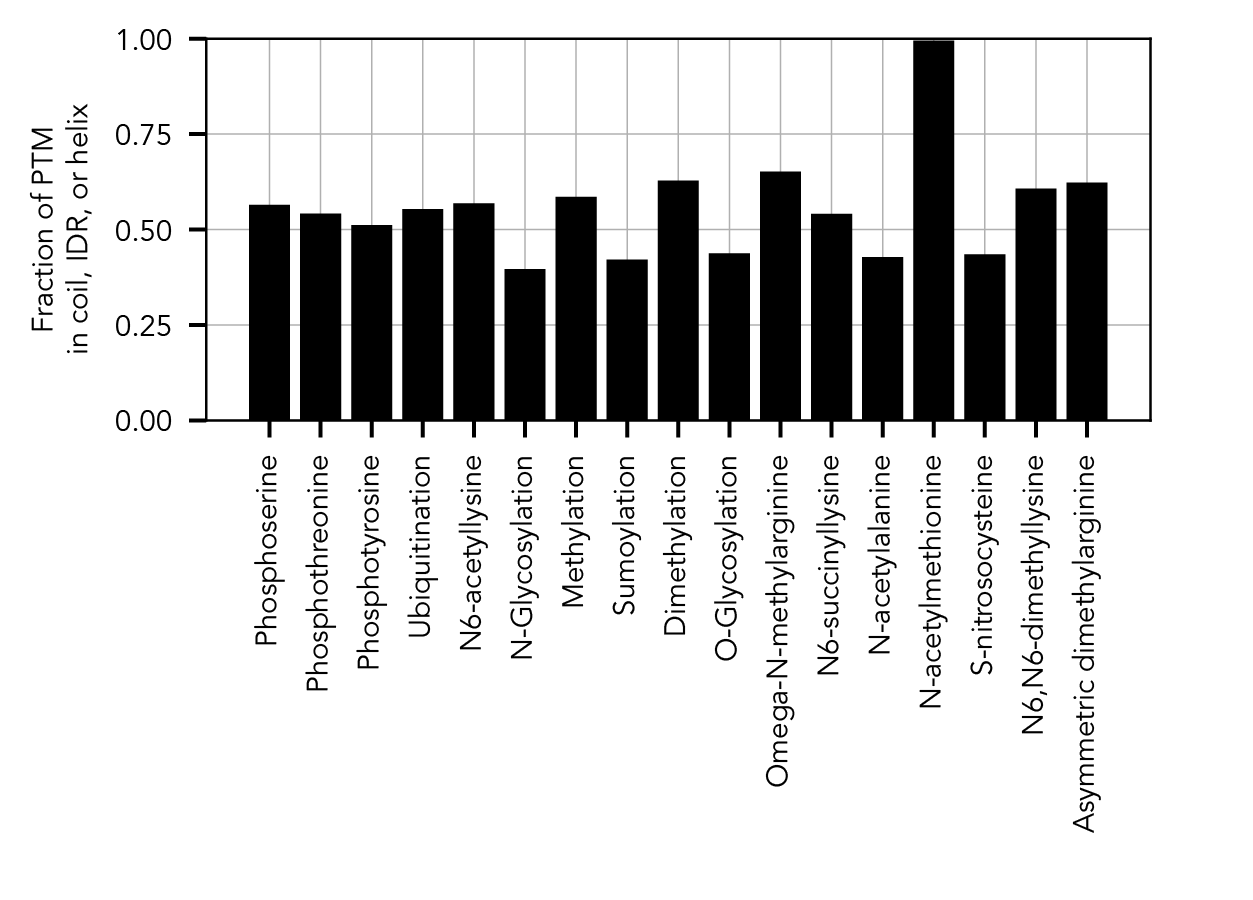


**Figure S2. Fraction of PTMs in solvent-accessible structural contexts.** For each type of modification, we determined what fraction of that residue was found in one of (1) unstructured/unclassified regions, (2) disordered regions, or (3) helices. For most PTMs, the majority were found in one of these three structural contexts.


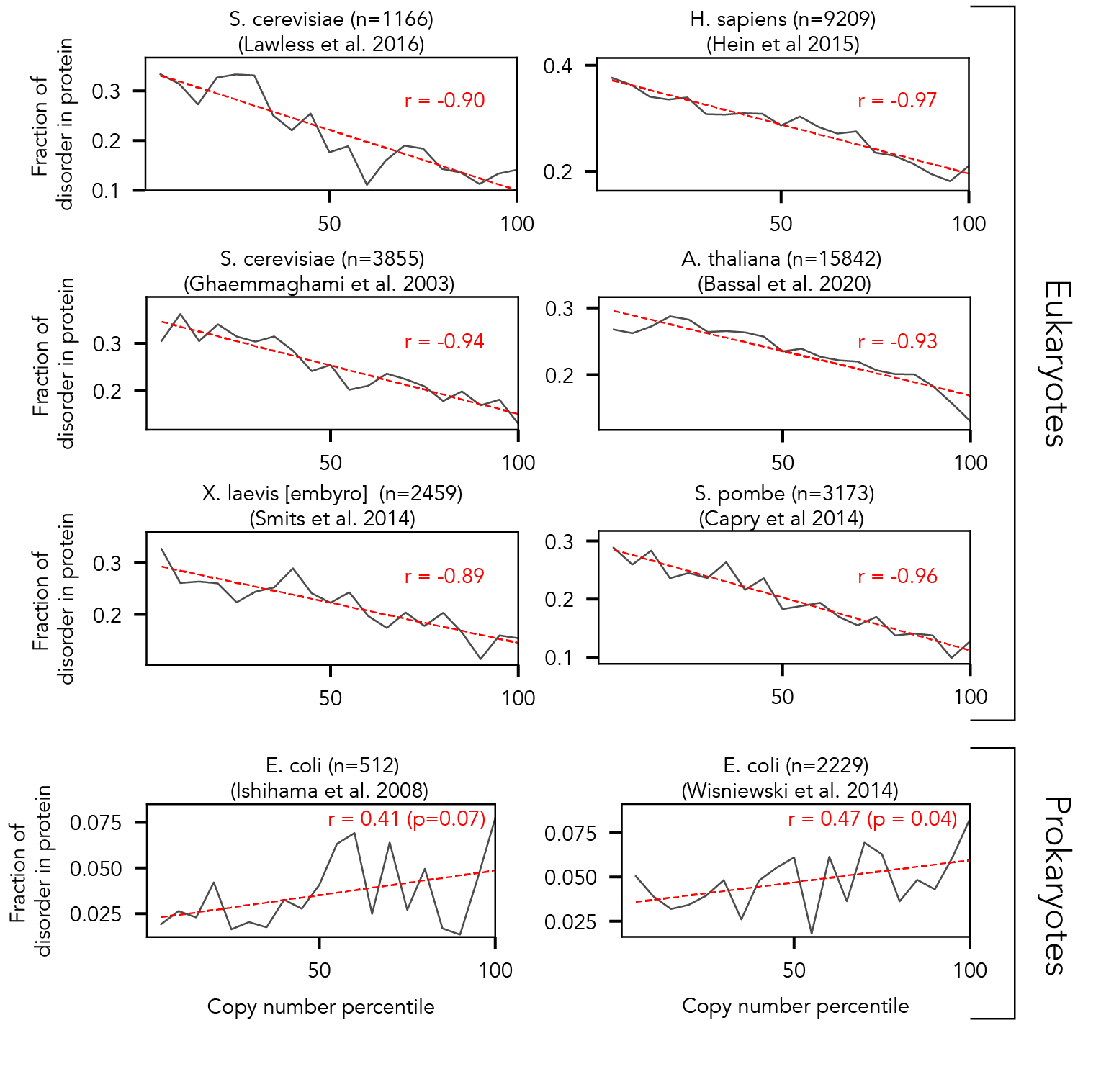


**Figure S3. Copy number vs. the average fraction of disorder across eight different mass spectrometry datasets**. Taking supplementary information from eight different studies, we correlated the average fraction of protein disorder in proteins within each ventile against the copy number ventiles (represented as percentiles for convenience), with correlations calculated using Pearson’s correlation coefficient. While the eukaryotes examined consistently show a negative correlation between copy number and the fraction of disorder, prokaryotes show no statistically significant trends (p > 0.01), a result that likely reflects the comparably lower propensity of disorder in prokaryotes.

**
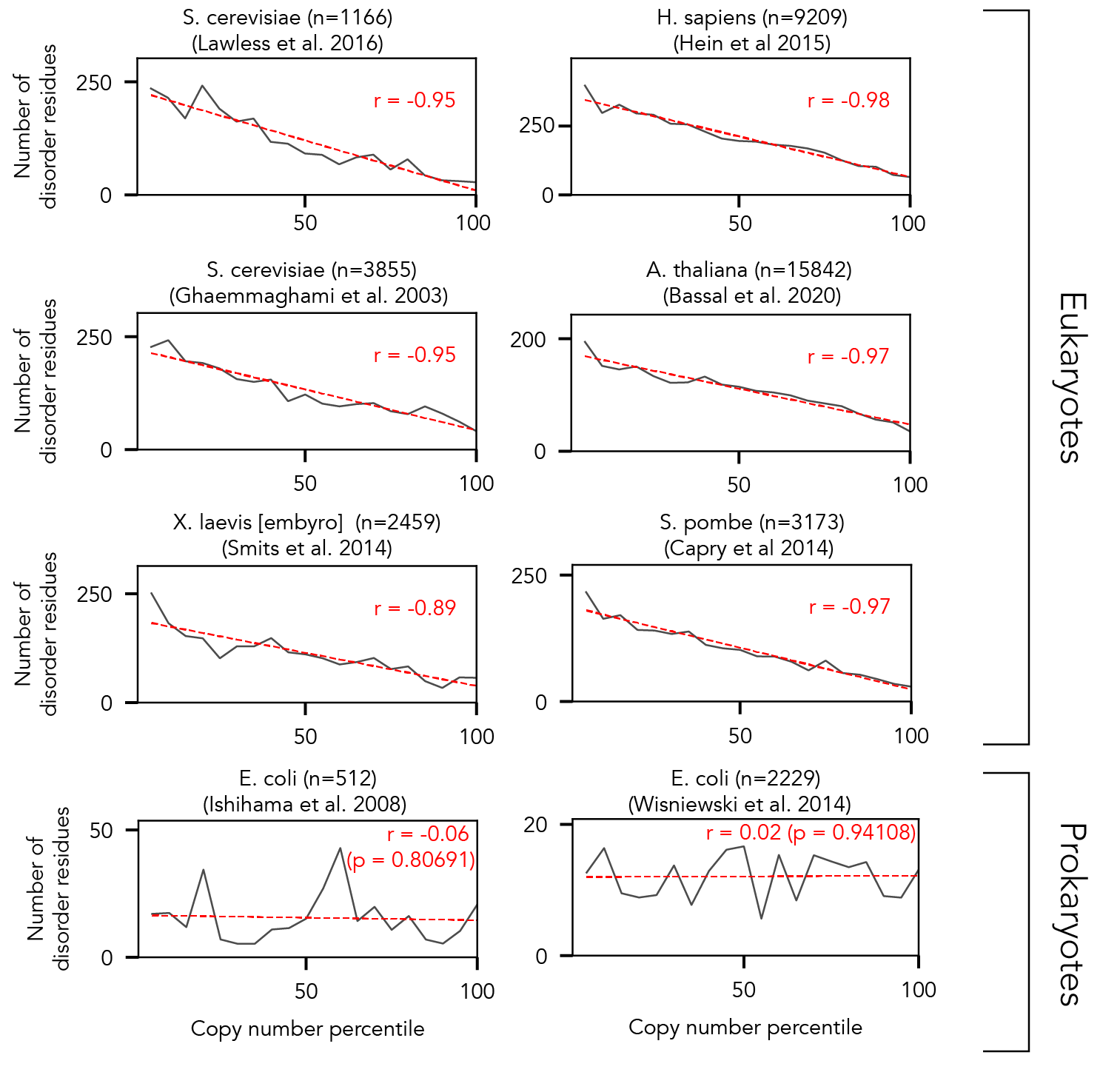
**

**Figure S4. Copy number vs. the average number of disorder residues across eight different mass spectrometry datasets**. Taking supplementary information from eight different studies, we correlated the average number of disorder residues in proteins within each ventile against the copy number ventiles (represented as percentiles for convenience), with correlations calculated using Pearson’s correlation coefficient. While the eukaryotes examined consistently show a negative correlation between copy number and number of disordered residues, prokaryotes show no statistically significant trends (p > 0.01), a result that likely reflects the comparably lower propensity of disorder in prokaryotes.

**
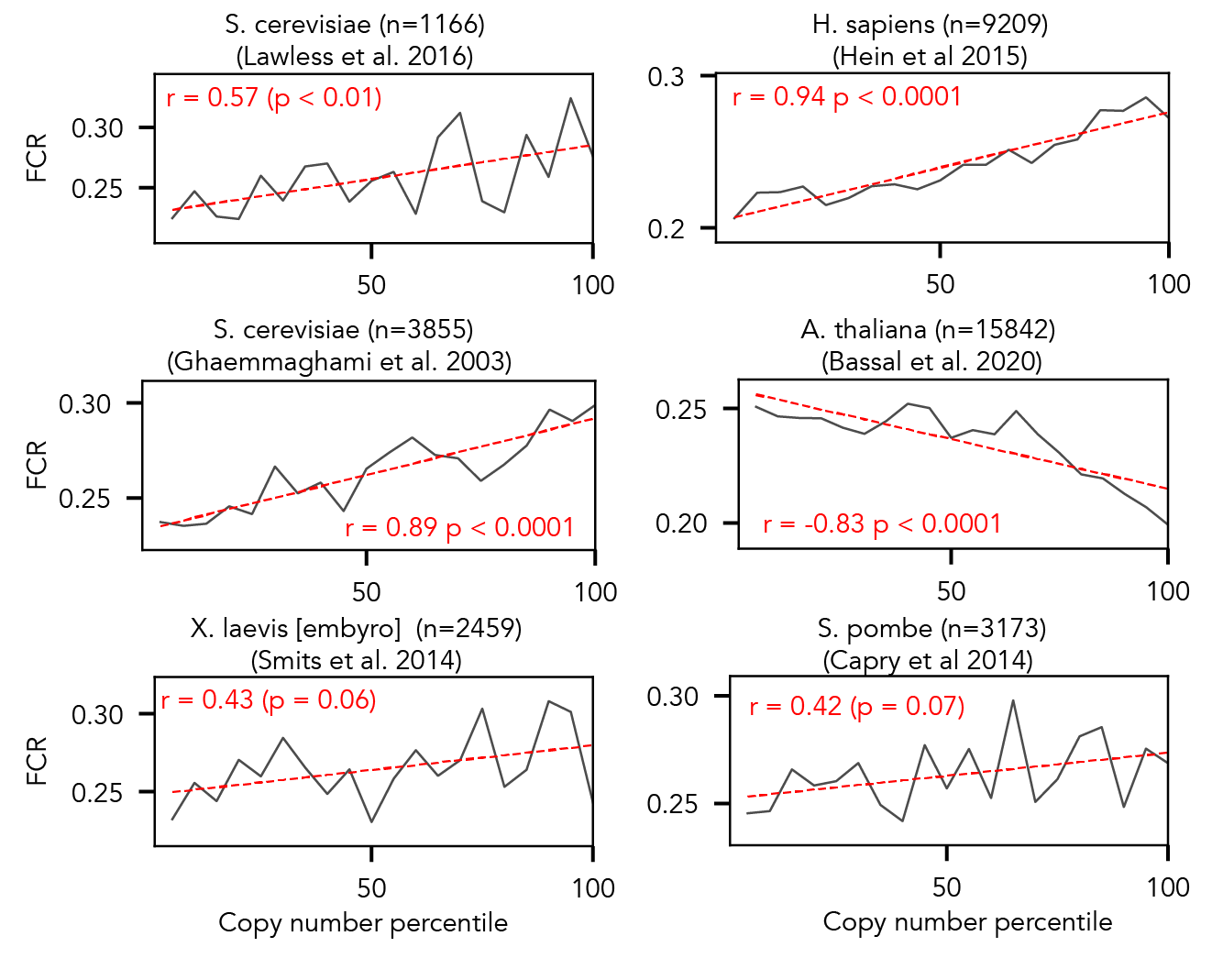
**

**Figure S5. Copy number vs. the mean fraction of charged residues (FCR) in disordered regions from eight different mass spectrometry datasets**. Taking supplementary information from eight different studies, we correlated the average FCR for disordered regions within each ventile against the copy number ventiles (represented as percentiles for convenience), with correlations calculated using Pearson’s correlation coefficient. Intriguingly, while yeast and human proteomes showed a consistent correlation whereby IDRs from highly-expressed proteins are more charged, this trend was not statistically significant in *X. laevis* or S. pombe, and a strong anticorrelation was observed in *A. thaliana*. The *E. coli* datasets had insufficient numbers of IDRs for this analysis to be performable.

**
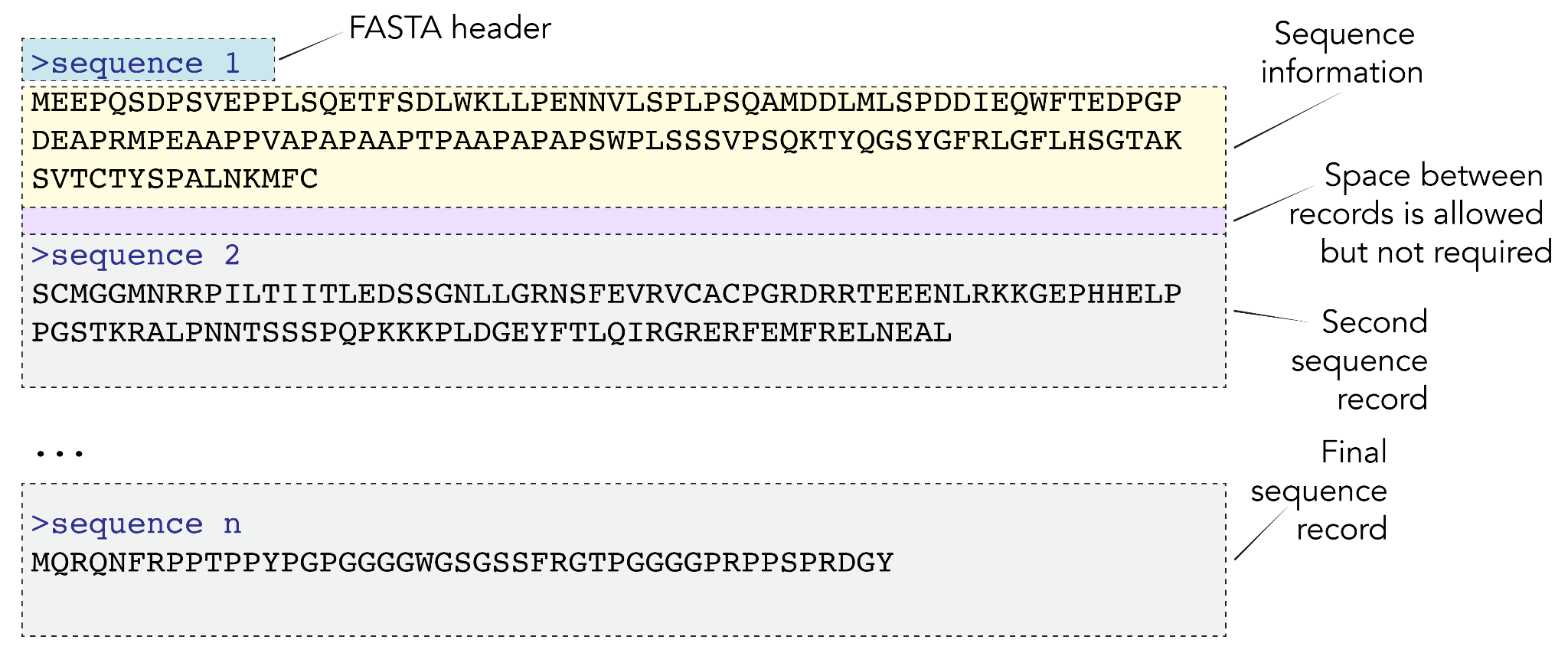
**

**Figure S6. Schematic of a FASTA file.** FASTA files are a standard format for sharing sequence information. The standard involves a header line (that starts with a “>” symbol) which ideally defines a unique piece of information that reports on the associated sequence. Below the header are one or more lines that contain the protein sequence. The protein record is ended either by the presence of the next header line or by the end of the file.

**SUPPLEMENTARY REFERENCES**

1. Harris, C. R., Millman, K. J., van der Walt, S. J., Gommers, R., Virtanen, P., Cournapeau, D., Wieser, E., Taylor, J., Berg, S., Smith, N. J., Kern, R., Picus, M., Hoyer, S., van Kerkwijk, M. H., Brett, M., Haldane, A., Del Río, J. F., Wiebe, M., Peterson, P., Gérard-Marchant, P., Sheppard, K., Reddy, T., Weckesser, W., Abbasi, H., Gohlke, C. & Oliphant, T. E. Array programming with NumPy. *Nature* **585,** 357–362 (2020).

2. van der Walt, S., Colbert, S. C. & Varoquaux, G. The NumPy Array: A Structure for Efficient Numerical Computation. *Computing in Science Engineering* **13,** 22–30 (2011).

3. Holehouse, A. S. *protfasta*. (2020). doi:[10.5281/zenodo.3862728](http://dx.doi.org/10.5281/zenodo.3862728)

4. Yanai, I. & Lercher, M. Night science. *Genome Biol.* **20,** 179 (2019).

5. UniProt, Consortium. UniProt: a hub for protein information. *Nucleic Acids Res.* **43,** D204–12 (2015).

6. Jumper, J., Evans, R., Pritzel, A., Green, T., Figurnov, M., Ronneberger, O., Tunyasuvunakool, K., Bates, R., Žídek, A., Potapenko, A., Bridgland, A., Meyer, C., Kohl, S. A. A., Ballard, A. J., Cowie, A., Romera-Paredes, B., Nikolov, S., Jain, R., Adler, J., Back, T., Petersen, S., Reiman, D., Clancy, E., Zielinski, M., Steinegger, M., Pacholska, M., Berghammer, T., Bodenstein, S., Silver, D., Vinyals, O., Senior, A. W., Kavukcuoglu, K., Kohli, P. & Hassabis, D. Highly accurate protein structure prediction with AlphaFold. *Nature* **596,** 583–589 (2021).

7. Tunyasuvunakool, K., Adler, J., Wu, Z., Green, T., Zielinski, M., Žídek, A., Bridgland, A., Cowie, A., Meyer, C., Laydon, A., Velankar, S., Kleywegt, G. J., Bateman, A., Evans, R., Pritzel, A., Figurnov, M., Ronneberger, O., Bates, R., Kohl, S. A. A., Potapenko, A., Ballard, A. J., Romera-Paredes, B., Nikolov, S., Jain, R., Clancy, E., Reiman, D., Petersen, S., Senior, A. W., Kavukcuoglu, K., Birney, E., Kohli, P., Jumper, J. & Hassabis, D. Highly accurate protein structure prediction for the human proteome. *Nature* **596,** 590–596 (2021).

8. Emenecker, R. J., Griffith, D. & Holehouse, A. S. Metapredict: a fast, accurate, and easy-to-use predictor of consensus disorder and structure. *Biophys. J.* **120,** 4312–4319 (2021).

9. Emenecker, R. J., Griffith, D. & Holehouse, A. S. Metapredict V2: An update to metapredict, a fast, accurate, and easy-to-use predictor of consensus disorder and structure. *bioRxiv* 2022.06.06.494887 (2022). doi:[10.1101/2022.06.06.494887](http://dx.doi.org/10.1101/2022.06.06.494887)

10. Gutierrez, J. I., Brittingham, G. P., Karadeniz, Y., Tran, K. D., Dutta, A., Holehouse, A. S., Peterson, C. L. & Holt, L. J. SWI/SNF senses carbon starvation with a pH-sensitive low-complexity sequence. *Elife* **11,** (2022).

11. Matlock, M. K., Holehouse, A. S. & Naegle, K. M. ProteomeScout: a repository and analysis resource for post-translational modifications and proteins. *Nucleic Acids Res.* **43,** D521–30 (2015).

12. Holehouse, A. S. & Naegle, K. M. Reproducible Analysis of Post-Translational Modifications in Proteomes--Application to Human Mutations. *PLoS One* **10,** e0144692 (2015).

13. Nagy, G. & Oostenbrink, C. Dihedral-based segment identification and classification of biopolymers I: proteins. *J. Chem. Inf. Model.* **54,** 266–277 (2014).

14. Nagy, G., Igaev, M., Jones, N. C., Hoffmann, S. V. & Grubmüller, H. SESCA: Predicting Circular Dichroism Spectra from Protein Molecular Structures. *J. Chem. Theory Comput.* **15,** 5087–5102 (2019).

15. Kabsch, W. & Sander, C. Dictionary of protein secondary structure: pattern recognition of hydrogen-bonded and geometrical features. *Biopolymers* **22,** 2577–2637 (1983).

16. McGibbon, R. T., Beauchamp, K. A., Harrigan, M. P., Klein, C., Swails, J. M., Hernández, C. X., Schwantes, C. R., Wang, L.-P., Lane, T. J. & Pande, V. S. MDTraj: a modern, open library for the analysis of molecular dynamics trajectories. *Biophys. J.* **109,** 1528–1532 (2015).

17. Thomas, P. D., Ebert, D., Muruganujan, A., Mushayahama, T., Albou, L.-P. & Mi, H. PANTHER: Making genome-scale phylogenetics accessible to all. *Protein Sci.* **31,** 8–22 (2022).

18. Hein, M. Y., Hubner, N. C., Poser, I., Cox, J., Nagaraj, N., Toyoda, Y., Gak, I. A., Weisswange, I., Mansfeld, J., Buchholz, F., Hyman, A. A. & Mann, M. A human interactome in three quantitative dimensions organized by stoichiometries and abundances. *Cell* **163,** 712–723 (2015).

19. Bassal, M., Abukhalaf, M., Majovsky, P., Thieme, D., Herr, T., Ayash, M., Tabassum, N., Al Shweiki, M. R., Proksch, C., Hmedat, A., Ziegler, J., Lee, J., Neumann, S. & Hoehenwarter, W. Reshaping of the Arabidopsis thaliana Proteome Landscape and Co-regulation of Proteins in Development and Immunity. *Mol. Plant* **13,** 1709–1732 (2020).

20. Bonnet, J., Lindeboom, R. G. H., Pokrovsky, D., Stricker, G., Çelik, M. H., Rupp, R. A. W., Gagneur, J., Vermeulen, M., Imhof, A. & Müller, J. Quantification of Proteins and Histone Marks in Drosophila Embryos Reveals Stoichiometric Relationships Impacting Chromatin Regulation. *Dev. Cell* **51,** 632–644.e6 (2019).

21. Wiśniewski, J. R. & Rakus, D. Quantitative analysis of the Escherichia coli proteome. *Data Brief* **1,** 7–11 (2014).

22. Ishihama, Y., Schmidt, T., Rappsilber, J., Mann, M., Hartl, F. U., Kerner, M. J. & Frishman, D. Protein abundance profiling of the Escherichia coli cytosol. *BMC Genomics* **9,** 102 (2008).

23. Carpy, A., Krug, K., Graf, S., Koch, A., Popic, S., Hauf, S. & Macek, B. Absolute proteome and phosphoproteome dynamics during the cell cycle of Schizosaccharomyces pombe (Fission Yeast). *Mol. Cell. Proteomics* **13,** 1925–1936 (2014).

24. Ghaemmaghami, S., Huh, W.-K., Bower, K., Howson, R. W., Belle, A., Dephoure, N., O’Shea, E. K. & Weissman, J. S. Global analysis of protein expression in yeast. *Nature* **425,** 737–741 (2003).

25. Lawless, C., Holman, S. W., Brownridge, P., Lanthaler, K., Harman, V. M., Watkins, R., Hammond, D. E., Miller, R. L., Sims, P. F. G., Grant, C. M., Eyers, C. E., Beynon, R. J. & Hubbard, S. J. Direct and Absolute Quantification of over 1800 Yeast Proteins via Selected Reaction Monitoring. *Mol. Cell. Proteomics* **15,** 1309–1322 (2016).

26. Lancaster, A. K., Nutter-Upham, A., Lindquist, S. & King, O. D. PLAAC: a web and command-line application to identify proteins with prion-like amino acid composition. *Bioinformatics* **30,** 2501–2502 (2014).

27. Alberti, S., Halfmann, R., King, O., Kapila, A. & Lindquist, S. A systematic survey identifies prions and illuminates sequence features of prionogenic proteins. *Cell* **137,** 146–158 (2009).
